# The Heterogeneity and Conditional Effects of Achievement Drivers: A Logistic Regression Analysis Based on Mathematics Scores of Portuguese Middle School Students

**DOI:** 10.3390/bs16101737

**Published:** 2026-09-24

**Authors:** Tuxian Zuo, Hongfeng Zhang, Liwei Ding

**Affiliations:** Faculty of Humanities and Social Sciences, Macao Polytechnic University, Macao, China

**Keywords:** academic performance prediction, mother’s educational level, logistic regression, generalized random forest, heterogeneous conditional associations

## Abstract

Identifying students at risk of academic failure remains a central challenge for education systems. Existing research assumes linear and homogeneous effects of family capital, overlooking context-dependent resource transformation. This study integrates Conservation of Resources Theory (COR) with the Process–Person–Context–Time (PPCT) model to propose that maternal education functions as a conditional resource, with its impact contingent on transformation through proximal processes like family interactions. Using data from 399 Portuguese students, logistic regression and generalized random forests were employed. Logistic regression identifies mid-term performance as the strongest predictor, family relationship quality as protective, and attendance and failure history as risk factors. Generalized random forest estimation reveals a non-significant average association of maternal education, yet conditional estimates fluctuate from −0.14 to 0.47, a pattern tentatively consistent with gain and loss spirals. These findings offer preliminary evidence for resource conditionality, are consistent with a conditional-resource interpretation of family capital effects, and generate hypotheses for future testing.

## 1. Introduction

Previous research investigating factors influencing academic performance has long been constrained by the linear reductionist paradigm. One approach focuses on static structural indicators such as family socioeconomic status and parental education levels (Şengönül, 2022), assuming that the impact of family capital is universal and homogeneous; the other emphasizes overt behavioral variables like time investment in learning and attendance rates (Xu et al., 2021), attempting to predict academic performance through a single behavioral variable. Although these two research approaches have yielded substantial findings, they share three fundamental limitations: first, they overlook the bioecological model. Specifically, they overlook the systemic principle of integrating the “process–context–time” framework; second, excessive reliance on cross-sectional data and correlation analyses makes it difficult to establish robust causal inferences (Cunningham, 2021); third—and most fundamentally—the implicit assumption that all influencing factors exhibit homogeneous effects across individuals, meaning a single factor exerts identical direction and intensity effects on all students (Rich & Owens, 2023). This assumption contradicts fundamental observations in educational practice: similar family capital investments may yield vastly different academic outcomes among students. Therefore, current research urgently needs to shift theoretically from single-factor explanations to systematic integration (Wang, 2023), and methodologically, from correlation-based predictions to heterogeneous causal inference (Couronné et al., 2018). Ignoring this heterogeneity may lead to biases and ineffectiveness in educational interventions (Bañeres et al., 2020).

The aforementioned paradigm is particularly evident in its focus on the question of “how mothers’ educational attainment influences their children’s academic performance.” Although maternal education is regarded as a core condition for children’s developmental capital (Davis-Kean et al., 2020), a recent OECD report on Portuguese higher education further supports that the influence of parental educational capital on children’s academic achievement exhibits significant contextual dependence (OECD, 2025), with its effect size being moderated by multiple factors such as school quality and access to information, indicating that its impact is not constant. However, such studies remain at the descriptive level of identifying “which factors moderate the magnitude of the effect,” failing to address two critical questions: Through what mechanisms does maternal education exert its influence? And why do results differ so markedly at comparable educational levels? To address these two questions, it is necessary to introduce a theoretical framework that simultaneously explains both the “conversion pathway” and the “conversion direction.” The integration of Conservation of Resources Theory and the bioecological model precisely provides such a framework: the former reveals the gain–loss spiral in resource dynamics (Bakker & de Vries, 2021), while the latter emphasizes that “proximal processes” constitute the core mechanism of development outcomes (Eriksson et al., 2018). The two theories complement each other: the Process–Person–Context–Time model identifies the pathway (Vélez-Agosto et al., 2017), while the Conservation of Resources Theory specifies the direction (Hobfoll et al., 2018), forming a coherent logical framework. Mother’s education, as a conditional resource, exerts its effect depending on whether it can be transformed through the proximal process of family interaction, the direction of this transformation being determined by the quality of that process. Within this framework, family relationship quality is operationalized as emotional support between parents and children, the effectiveness of communication, and the frequency of conflicts (Mastrotheodoros et al., 2020), serving both as a proximal process and an energy resource. Cross-cultural studies further indicate that parent–child conflicts weaken the educational impact of parental involvement, particularly in highly educated families (Marsh et al., 2023). It suggests that whether a mother’s educational capital translates into academic advantages depends not on the capital itself, but on the quality of its transmission channel—the “family emotional environment” (Baptist & Hamon, 2022).

Beyond familial factors, the cumulative effect of learning behaviors also constitutes a significant confounding source. Mid-term academic performance encapsulates the cumulative impact of prior learning behaviors and serves as the strongest predictor of final outcomes (Kesgin et al., 2025). A nonlinear relationship between learning time and academic achievement has been suggested in prior reviews (Guo et al., 2024), though this pattern was not supported in the present data after covariate adjustments. Failure to control for these variables may obscure or distort the true effects of maternal education due to omission bias. Therefore, a rigorous causal inference framework must incorporate learning behavioral variables as control factors, enabling a clear estimation of the heterogeneous causal effects of maternal education—a conditional resource—by eliminating the cumulative effects of behavior.

This study focuses on Portuguese secondary school students, primarily based on the following considerations. First, Portugal’s PISA mathematics scores are near the OECD average and have been steadily rising (Liu et al., 2024), providing sufficient score variations to examine the moderating effects of heterogeneous factors such as family capital. Second, Portugal exhibits significant intergenerational educational disparities, with paternal education showing a strong predictive effect on mathematics performance (Clemente-Casinhas et al., 2025), aligning with the cultural tradition in Southern Europe that emphasizes the paternal role (Cruz et al., 2020). Portugal provides an ideal sociocultural context for testing the resource-complementary hypothesis of paternal education as a compensatory mechanism for maternal educational investment. Research findings from such contexts hold comparable relevance for Latin American regions sharing the Southern European cultural sphere and similar family structures.

## 2. Research Design

### 2.1. Research Hypotheses

Based on the aforementioned integrated framework, this study proposes three core hypotheses: H1 (Conditional Hypothesis on Resource Transformation): The average association of maternal education level with mathematics scores is expected to be heterogeneous across family contexts; when averaged over the full sample, it may be attenuated and indistinguishable from zero because the effect of conditional resources depends on the quality of proximal processes; the average effect divorced from context lacks theoretical validity. Significant associations, if any, are hypothesized to emerge only within specific subgroups defined by family relationship quality and paternal education.

H2 (Gain and Loss Spiral Hypothesis): H2a: In subgroups with higher family relationship quality, maternal education exhibits a positive conditional average treatment effect, facilitating smooth proximal processes and channeling resources into a gain cycle; H2b: Among students with high maternal education, lower family relationship quality is associated with a negative CATE, obstructing proximal processes and giving rise to the “family education paradox.”

H3 (Resource Complementarity Hypothesis): In the subgroup with lower maternal education levels, higher paternal education levels generate a compensatory positive effect, reflecting both the resource substitution logic of the COR theory and the multi-layered system complementarity logic of the PPCT model.

Based on three hypotheses, we pose three questions: (1) What are the key predictors of mathematics performance? (2) Does the causal effect of maternal education level follow the patterns predicted by H1, H2a, H2b, and H3? (3) What practical implications do these findings have for identifying academic risks?

### 2.2. Overview of Analysis Strategies

Based on the research hypothesis, a two-stage analytical approach is employed to achieve the dual objectives of prediction and causal inference. The first stage involves predictive analysis: a logistic regression model is constructed to identify key predictors of academic success among students and quantify the direction and magnitude of each factor’s association. This stage addresses Research Question 1 and provides interpretable baseline results for subsequent causal analysis.

The second stage involves causal heterogeneity analysis. Using maternal education level as the treatment variable, a generalized random forest model is employed to estimate its heterogeneous causal effects. This stage tests three hypotheses—H1, H2a, H2b, and H3—to address Research Question 2. Both stages utilize the same dataset but focus on different analytical objectives: the former emphasizes prediction, while the latter focuses on causality. Figure 1 illustrates the complete analytical roadmap.

### 2.3. Data Sources and Sample

This study utilized the Portuguese secondary school mathematics learning dataset, a publicly available dataset from the UCI Machine Learning Repository (Cortez & Silva, 2008), originally collected from two public schools in Portugal. The dataset comprises 33 features from 399 students. The Portuguese education system employs a phased assessment framework, comprising initial scores (G1), mid-term scores (G2), and final summative scores (G3), providing a data foundation for tracking academic progression (Ojajuni et al., 2021). We confirmed no missing values across all 33 variables (N = 399 complete cases); therefore, no imputation was required. Continuous variables were standardized using the Z-score transformation. Categorical variables were converted to factor format for automatic dummy variable generation in the logistic regression model. Hierarchical sampling was employed to divide the training and testing datasets in an 8:2 ratio, ensuring consistent pass rate distributions between the two groups. The pass rate was 66.3% for the training set and 67.1% for the testing set.

### 2.4. Variable Definition and Measurement

The operational definitions of the core variables and their positioning within the theoretical framework are presented in Table 1. Both maternal and paternal education levels are coded on a scale of 1 to 5, with higher scores indicating higher educational attainment. Family relationship quality is also coded on a scale of 1 to 5, reflecting the level of emotional support and communication between parents and children. Initial scores (G1), mid-term scores (G2), and final scores (G3) all range from 0 to 20 points. The academic pass variable (Pass) is defined as a G3 score of 10 or higher; the definition for mathematics is as follows in Formula (1):

(1) $$Pass=\left\{\begin{array}{c} 1\;if\;G3\geq 10\\ 0\;\;\;otherwise \end{array}\right.$$

From a theoretical perspective, a mother’s educational level constitutes a conditional resource within the Conservation of Resources Theory, with its effects dependent on transformation pathways; family relationship quality serves dual roles: as both a proximal process in the Process–Person–Context–Time model and an energy resource in the COR Theory; attendance frequency and academic failure history serve as indicators of resource depletion; while initial and mid-term academic performance represent nodes for resource accumulation.

## 3. Methodology

### 3.1. Exploratory Data Analysis

Prior to formal modeling, this study employed exploratory data analysis to achieve three objectives: verifying whether the distribution of continuous variables met logistic regression assumptions, identifying potential nonlinear relationships, and providing descriptive references for interpreting heterogeneity.

First, the ggpairs function was employed to construct the variable relationship matrix (Sohil et al., 2022): the lower triangle displays a scatter plot to identify nonlinear patterns and outliers; the upper triangle shows correlation coefficients; the diagonal presents kernel density curves to analyze the distribution pattern of scores (Scrucca et al., 2023), with particular emphasis on symmetry testing. Second, the Pearson correlation coefficient was calculated and plotted as a heat map (Ahlmann-Eltze & Patil, 2021), using color intensity to represent correlation strength. This approach serves two purposes: identifying correlations with the dependent variable and the most significant leading indicator, and the preliminary identification of highly correlated pairs among independent variables, which serve as the basis for subsequent multicollinearity tests.

To address the linear limitations of the Pearson correlation coefficient, this study further conducted grouped visual analyses. These included plotting pass rate histograms with error bars after stratifying learning durations to identify any inverted U-shaped relationships; generating violin plots comparing academic performance distributions across three family relationship quality groups; grouping attendance absences by quartiles and calculating the 95% confidence interval for pass rates to detect potential threshold effects; and constructing partial dependence plots for parental education levels after controlling for residual variables to examine whether their marginal effects exhibited monotonic, saturated, or non-monotonic characteristics.

The aforementioned three-step exploratory analysis systematically evaluated variable distribution, linear strength, and nonlinear signals. Its findings directly guide variable encoding strategies in logistic regression and provide a traceable descriptive basis for subgroup division in the conditional averaging treatment effects of generalized random forests, ensuring that model specifications align with actual data characteristics.

### 3.2. Logistic Regression Model

Logistic regression is suitable for binary classification problems, providing the coefficients of variables and the odds ratio, which clearly reveal the direction and strength of each factor’s association with the pass probability. The model represents the relationship between student pass probability and the predictor variables, as specified in Formula (2):

(2) $$\log(\frac{P(Pass=1)}{1-P(Pass=1)})=\beta_{0}+\sum_{i=1}^{n}\beta_{i}X_{i}$$

Here, coefficient β represents the change in the log-odds (logit) for each one-unit increase in the dependent variable *X*, reflecting both the direction and magnitude of the factor’s association with students’ probability outcomes (Chung, 2020). The model employs 5-fold cross-validation to prevent overfitting and evaluates performance using metrics such as accuracy (Probst et al., 2019), AUC, sensitivity, and specificity. The variance inflation factor test shows that all predictor variables have VIF values ranging from 1.01 to 3.76, all below the empirical threshold of 5, indicating no significant multicollinearity in the model.

To ensure the robustness of the model, this study conducted a multi-dimensional sensitivity analysis. Sample split sensitivity analysis was used to examine the degree to which the model’s performance depends on the specific partitioning of training and testing sets. Variable inclusion sensitivity analysis was performed by removing each predictor variable one at a time and observing the resulting changes in model accuracy in order to identify core predictive factors. In addition, repeated validation was carried out using different random seeds to test the stability of the estimation results. To evaluate the model’s utility as a genuinely early warning tool, we conducted a sensitivity analysis by fitting a reduced model (Model B) excluding G1 and G2, retaining only demographic, family, and behavioral variables available at the beginning of the academic term. Model performance was compared with the full model using 5-fold cross-validated ROC-AUC, and the DeLong test was applied to assess the statistical significance of the AUC difference.

### 3.3. Analysis of Heterogeneous Conditional Effects

Figure 2 presents the DAG for causal identification, specifying Medu as treatment, G3 as outcome, and G1/G2 as mediators. Following this structure, G1 and G2 were excluded from the covariate adjustment.

Based on Figure 2, this research employs the potential outcomes framework (Colnet et al., 2024) and the generalized random forest model (Athey et al., 2019) to further investigate the causal relationships and heterogeneity among variables and to test the hypotheses H1, H2, and H3 proposed in this study. Under the potential outcomes framework, each student has two potential states, as expressed in Formula (3): the academic achievement under a high level of maternal education, and that under a low level of maternal education. The difference between these two states constitutes the individual treatment effect. Averaging the individual treatment effects across all students yields the average treatment effect. When estimated under specific family characteristics, the conditional average treatment effect is obtained. The formulas for these effects are as follows:

Potential outcomes framework:

(3) $$Y_{i}=Y_{i}^{\left(1\right)}\cdot Medu_{i}+Y_{i}^{\left(0\right)}\cdot (1-Medu_{i})$$

Averaging the individual treatment effects across all students yields the average treatment effect (ATE), as shown in Formula (4):

(4) $$ATE=E\left[Y^{\left(1\right)}-Y^{\left(0\right)}\right]$$

When estimated under specific family characteristics, the conditional average treatment effect (CATE) is obtained, as presented in Formula (5):

(5) $$CATE(Medi)=E\left[Y_{i}^{\left(1\right)}-Y_{i}^{\left(0\right)}\left|Medu_{i}=Medi\right.\right]$$

Here, $Y_{i}(W)$ denotes the latent mathematics score of student *i* given the mother’s education level $W_{i}=W$, where *i* represents the individual student, $W_{i}$ is the treatment variable for the mother’s education level, and *W* is the specific value of the education level; *E* represents the expected value, used to calculate the average effect at the population level. Based on this framework, a generalized random forest was employed for causal inference. Maternal education is a 5-point ordinal variable, treated as a continuous treatment in the generalized random forest framework. A one-unit increase corresponds to a one-category increment, so the estimated CATE is the expected change in G3 (0–20 points) per level increase in maternal education, holding other covariates constant. This assumes equal intervals between adjacent ordinal categories—a common simplification for interpretability, with limitations acknowledged in Section 6.2. Following the DAG (Figure 2), the model controlled for pre-treatment covariates while excluding mediators G1 and G2. The model was implemented using causal_forest. The built-in orthogonalization technique adjusts for measured confounding in predictions for both treatment and outcome variables, yielding estimates that are less sensitive to the specification of the outcome and treatment models under the assumption of no unmeasured confounding (Nie & Wager, 2021). This effectively mitigates estimation biases inherent in traditional regression models due to missing variables or model misspecification (Géron, 2019). The generalized random forest simultaneously produces ATE and CATE, revealing the differentiated impact of maternal education levels across various family environment subgroups and overcoming the homogeneity assumption of conventional regression models (Nemțoc, 2025).

Regarding hyperparameter settings, the model specifies 2000 trees and enables automatic hyperparameter optimization via the ‘tune.parameters = “all”’ parameter, which automatically selects the optimal hyperparameter combination through cross-validation, eliminating the subjectivity associated with manual parameter tuning. To validate the reliability of causal effect estimates, this study constructed confidence intervals using Monte Carlo simulations and performed multiple estimations with different random seeds to assess result stability. The results demonstrate that the model’s estimates exhibit high robustness.

## 4. Result Analysis

### 4.1. Data Distribution and Analysis of Key Associated Factors

The distribution pattern of final scores G3 is shown in Figure 3, exhibiting a typical bimodal distribution with the main peak at 10 points and the secondary peak at 15 points, reflecting the clear differentiation between “passing” and “failing” students. Notably, 33% of students did not meet the score requirements, with 18% falling within the “critical range” of 8–10 points—a group representing a potential priority for educational intervention.

The distribution differences in academic performance between male and female students are shown in Figure 4. The median and mean scores of male students were higher than those of female students, with a more dispersed score distribution and a pronounced trend toward polarization. In contrast, female students’ scores were relatively concentrated, predominantly falling within the middle to lower score ranges.

The correlation matrix of the three examination scores is shown in Figure 5. The final score G3 exhibits a highly linear correlation with both the initial score G1 and the mid-term score G2. Multivariate regression analysis revealed that, after controlling for G1, the coefficient of influence of G2 on G3 was 0.985. In the univariate regression, G2’s R^2^ value was significantly higher than that of G1, supporting G2 as the strongest predictor of G3.

Figure 6 of the Pearson correlation coefficient heat map demonstrates a significant positive correlation between parental education levels and final academic performance. The linear relationship between family relationship quality and attendance frequency with academic performance was not statistically significant, suggesting that the relationship between these variables and academic performance may exhibit nonlinear or conditional characteristics.

The nonlinear relationship between study duration and academic performance is illustrated in Figure 7. Although exploratory data analysis suggested some descriptive variation in low-score proportions across study time categories, a formal nonlinearity test controlling for covariates found no statistically significant quadratic relationship between study time and passing probability (χ^2^(1) = 0.44, *p* = 0.509). The apparent descriptive pattern did not withstand covariate adjustment, suggesting it may reflect confounding—particularly from prior academic performance—rather than a genuine nonlinear effect. We therefore caution against strong practical recommendations regarding an optimal study duration based on these data.

The relationship between family relationship quality and academic performance is shown in Figure 8. No consistent correlation was observed between the mean and median scores across different family relationship rating groups, and each group included a certain proportion of students who did not meet the required standards.

### 4.2. Prediction Model Performance and Explanatory Analysis

The logistic regression model demonstrated excellent predictive performance on the test set. The model achieved an accuracy of 88.61%, significantly higher than the baseline no-information rate of 67.09%. The sensitivity was 86.79%, specificity 92.31%, precision 95.83%, and F1-score 0.911. The ROC-AUC was 0.895, indicating excellent discriminative ability. The model identified seven false negatives and two false positives (Chen & Zhai, 2023). The false negatives are particularly concerning for early-warning purposes, as these students were predicted to fail but actually passed. This suggests that some students may pass the examination due to temporary factors yet have an unstable academic foundation. Therefore, it is crucial to include such students in continuous monitoring to effectively mitigate the potential risk of underreporting in early-warning mechanisms. Figure 9 displays the estimated coefficient values of the logistic regression model, with the odds ratios for each variable reflecting their strength and direction of influence on the probability of the outcome (Lundberg et al., 2020).

The mid-term score G2 is the decisive factor in predicting the pass probability; each one-point increase in G2 multiplies the odds of passing by 7.06 (OR = 7.06). The initial score G1 also has a positive predictive effect, with each one-point increase multiplying the passing odds by threefold. Family relationship quality serves as an important protective factor; each grade level improvement multiplies the passing odds by 1.5 times. Regarding risk factors, each additional failure reduces the passing odds by 51%, while each additional 10 absences reduces the passing odds by 18%. All these associations were obtained after controlling for other variables. Because odds ratios are multiplicative on the odds scale, they should not be interpreted as direct changes in the probability of passing.

### 4.3. Heterogeneous Conditional Effects of Mother’s Educational Level

Following the DAG specification (Figure 2), G1 and G2 were excluded as covariates. This section tests hypotheses H1, H2a/H2b, and H3. The generalized random forest analysis indicates that the estimated average association of maternal education with mathematics scores is 0.12 points, with its 95% confidence interval containing zero, suggesting that among the overall student population, maternal education level does not exhibit a statistically significant direct linear association. This result is consistent with H1. However, estimates of the conditional average treatment effects reveal heterogeneous patterns masked by the overall effect. As shown in Table 2, the point estimates vary across different family contexts, ranging from −0.14 to 0.47, though most confidence intervals include zero. The direction and intensity of the effect are jointly moderated by the father’s educational level and the quality of family relationships.

The key patterns are as follows. First, the interaction between maternal education and family relationships is directionally consistent with the gain–loss spiral. When both are at high levels, the point estimate is positive and the only subgroup reaching statistical significance, consistent with H2a. However, when maternal education is high but family relationships are strained, the point estimate is negative—offering suggestive evidence consistent with H2b, though the wide confidence interval indicates this pattern requires further investigation. Second, the CATE estimates suggest a possible complementarity between parental educational resources. When maternal education is lower but paternal education is higher, the point estimate shows a moderate positive association, tentatively aligning with H3, although the confidence interval includes zero. When both maternal and paternal education levels are high, the effect does not exhibit a cumulative enhancement, indicating diminishing marginal returns or functional overlap between parental educational resources.

## 5. Discussion and Contributions

### 5.1. Discussion

#### 5.1.1. The Strong Predictive Role of Mid-Term Performance

The logistic regression results indicate that the mid-term score G2 is the strongest predictor of the final score, with each one-point increase in G2 multiplying the odds of passing by approximately 7.06. As noted in Section 4.2, this odds ratio is not a direct probability multiplier. While previous studies have primarily explained this phenomenon through statistical correlations (Namoun & Alshanqiti, 2021), our findings reveal deeper underlying mechanisms behind G2’s strong predictive power. Within Portugal’s secondary education system, G2 serves as a pivotal assessment criterion in institutional design, with its scores directly triggering formal interventions by teachers and parents (Ferrão & Almeida, 2019). G2 is defined as the resource accumulation node, whose exponential impact is consistent with the temporal dimension proposition of the Process–Person–Context–Time model: the academic capital accumulated through earlier proximal processes ultimately converges at the G2 time node, indicating whether students have surpassed critical competency milestones in their academic development (Yeager et al., 2019). This nonlinear characteristic supports the core logic of the Conservation of Resources Theory: mid-term performance serves as a key indicator of bifurcation in the resource circulation pathway—only 10% of students with a low G2 pass, whereas nearly all students with a high G2 pass. Therefore, action should be taken within this “opportunity window” at G2. This approach is most likely to alter academic trajectories, aligning closely with the early intervention philosophy emphasized by Portugal’s “School Success Plus” program, and also provides a theoretical foundation for a multi-level early-warning system. The sensitivity analysis excluding G1 and G2 supported that G2 is the dominant predictor. Thus, the model is best deployed after the G2 assessment rather than as a stand-alone early-warning tool.

#### 5.1.2. Gender Differences and Variability Differentiation

Male students’ mathematics scores average slightly higher than those of female students, with a more dispersed distribution that exhibits polarization; female scores, in contrast, cluster around the lower-middle range. This pattern can be understood at two levels. At the variance level, a greater score variability among males is a cross-cultural phenomenon (Reinhold et al., 2020). At the mean level, the higher average scores of males do not contradict the “gender equality paradox”. Stoet and Geary argue that societies with higher levels of gender equality often show larger mean differences—not denying the existence of such disparities (Stoet & Geary, 2018), but highlighting their sociocultural roots. The common driving force behind both lies in gender role expectations: the observed gender differences in score dispersion may be attributable to a range of sociocultural and psychological factors, but these mechanisms were not directly measured in this study. We therefore refrain from attributing these descriptive differences to specific behavioral strategies or proposing gender-specific interventions based solely on these observations.

#### 5.1.3. The Indirect Mechanism of Family Relationship Quality

Although exploratory data analysis did not reveal a direct linear correlation between family relationships and academic performance, the findings remained statistically significant in logistic regression, indicating that the underlying mechanisms are far more complex than direct effects. This discovery is consistent with the dual theoretical framework for family relationship quality: it serves both as a “proximal process” in the Process–Person–Context–Time model and as an “energy resource” within the Conservation of Resources Theory. Healthy family relationships do not directly translate into higher academic scores but exert their influence through a dual mechanism. On one hand, they buffer against risk factors such as academic stress or negative peer influences (L. Zhao & Zhao, 2022); on the other hand, they significantly enhance the effectiveness of other positive resources (Zhuo et al., 2022). Family interaction constitutes the most critical “proximal process” within the microsystem, and its quality is associated with whether other resources can be effectively utilized (Eriksson et al., 2018). Portuguese adolescents exhibit higher emotional sensitivity to family conflicts compared to those in Nordic countries, with family relationship quality showing a more pronounced predictive effect on academic adaptation (Botelho Guedes et al., 2022). This indicates that family relationships in Portugal serve not only as a moderating variable but also as a “core buffer” against academic risks. The findings of this study align with recent research on family support mechanisms: family support primarily exerts its influence indirectly through mediating or moderating pathways, rather than directly affecting academic performance (Estévez et al., 2021). Akçapınar’s study explicitly demonstrates that positive family relationships significantly enhance students’ psychological resilience (Akçapınar et al., 2019). The practical implication is that the value of family relationships should not be overlooked merely because their direct correlation with academic achievement appears insignificant—particularly in Southern European societies with strong family-oriented cultures. For students with strained family relationships, even if their mothers have high levels of education, the effectiveness of parenting may be significantly diminished (Park & Lee, 2020).

#### 5.1.4. The Conditional Effect of a Mother’s Educational Level

The generalized random forest estimation reveals that the estimated average association of maternal education with math scores is 0.12 points, with the 95% confidence interval containing zero, which is consistent with H1. The conditional average treatment effect estimation uncovers heterogeneous patterns masked by overall averaging: the effect of maternal education fluctuates sharply between −0.14 and 0.47, with family relationship quality serving as the primary driver. When high maternal education coexists with weak family relationships, the point estimate was negative. This pattern is broadly consistent with what prior theoretical work has termed the ‘family education paradox,’ but the wide confidence interval and unmeasured mediators mean this interpretation is speculative and requires replication. The expectations of mothers with high educational backgrounds may be perceived by their children as control and pressure (Tang & Jiang, 2024), transforming educational investment into a source that undermines learning motivation (Marsh et al., 2023). Similarly, Barrera-Osorio et al. (2020) found that in families with high educational backgrounds but lacking emotional support, school-home interventions may prove counterproductive. When low maternal education combines with high paternal education, the point estimate was moderately positive—a pattern suggestive of resource complementarity. Paternal education may function as external system capital that partially compensates for lower maternal education within the microsystem, though the confidence interval included zero. These findings highlight both the limitations and the substitutability among resources (Strømme & Wiborg, 2024). Notably, the point estimate for the subgroup with lower maternal but higher paternal education (0.47) was numerically larger than that for the dual-high group (0.20). However, because these two CATEs are defined at different starting levels of maternal education and estimated on different subsamples, their magnitudes are not directly comparable, and both confidence intervals include zero. We therefore refrain from interpreting this difference as evidence of diminishing marginal returns or functional overlap. This pattern is nonetheless consistent with the unique cultural capital transmission dynamics of paternal education in Portugal (Clemente-Casinhas et al., 2025) and with the broader Southern European family structure documented by Cruz et al. (2020).

#### 5.1.5. Study Time and Academic Performance

Although prior research has questioned whether ‘‘the longer one studies, the better’’ (Spitzer, 2022), our exploratory data analysis suggested only a descriptive variation in low-score proportions across study time categories. A formal likelihood ratio test comparing linear and quadratic specifications of study time, controlling for G1, G2, and other covariates, found no statistically significant nonlinear relationship. This null finding suggests that the apparent descriptive pattern may reflect confounding—particularly from prior academic performance—rather than a genuine nonlinear effect. We therefore caution against strong practical recommendations regarding an optimal study duration based on these data. Future studies with larger samples and experimental designs are needed to reliably test potential nonlinear effects of study time on academic achievement (Sweller, 2020).

#### 5.1.6. Model Performance and Early Warning Applications

The logistic regression model developed in this study achieved an accuracy rate of 88.61% on the test set, with its high sensitivity and specificity rivaling or even surpassing those of common black-box models reported in existing literature. More importantly, the model provides clearly interpretable coefficients that precisely reveal the direction and magnitude of association from each factor. These insights support the feasibility of multi-level early-warning systems for academic intervention and further identify key thresholds applicable for dynamic interventions, offering practical guidance for educators (Zhu & Yang, 2024). The false-negative cases identified during the study indicate that some students may pass the examination due to temporary factors yet have an unstable academic foundation. Therefore, it is crucial to include such students in continuous monitoring to effectively mitigate the potential risk of underreporting in early-warning mechanisms (Z. Zhao & Ren, 2025).

### 5.2. Contribution

First, the point estimates for the gain-spiral and loss-spiral subgroups were directionally consistent with H2a and H2b, respectively. For the loss-spiral subgroup, the point estimate was negative, but the confidence interval was wide and included zero; this pattern should be treated as hypothesis-generating rather than as evidence for a “family education paradox.” It is consistent with a conditional-resource interpretation of COR theory—resources are not unconditionally beneficial, and their valence may depend on the quality of proximal processes—but the present data cannot distinguish a true negative effect from sampling noise. When the family emotional environment is adverse, the cultural capital of highly educated mothers may transform from developmental resources into academic stressors, which may, in turn, initiate the loss spiral. Second, this study is consistent with the core proposition of the PPCT framework: the proximal process serves as the essential pathway for transforming resources into developmental outcomes, with the maternal education effect entirely dependent on the quality of family interactions. Third, the study identifies a compensation mechanism for paternal education relative to maternal education (H3), reflecting both the resource substitution logic of the COR theory and the complementary multi-layered system logic of the PPCT model. Fourth, by integrating the two theories, a division-of-labor framework emerges where “PPCT refers to the pathway, while COR indicates the direction”: the former specifies that resource transformation must occur through the proximal process, and the latter reveals that the transformation direction depends on process quality. This framework demonstrates that the family emotional environment is crucial for translating maternal education into academic advantages, serving as the fundamental prerequisite.

### 5.3. Recommendations

This study translates the key predictive factors identified by logistic regression into actionable hierarchical intervention tools, directly addressing Research Question 3. Using mid-term score G2 and initial score G1 as core indicators, and incorporating dynamic data such as course failure history and attendance records, a multi-level early-warning model was developed. Existing research has similarly supported the significant value of predictive models based on historical academic performance in educational interventions (Seetee et al., 2021), which, through longitudinal tracking of middle school students’ mathematics scores, found that second-semester performance exhibited the strongest predictive power for final grades—a finding highly consistent with this study’s conclusion that G2 is the strongest predictor. This finding supports the use of interim performance as a core early-warning indicator.

Based on the logistic regression model coefficients and risk factor analysis, this study proposes a three-tier early-warning framework using mid-term performance (G2) as the primary stratification indicator. Students with a G2 below 10 were classified as high risk (*n* = 70, pass rate = 10%), those with a G2 between 10 and 14 as medium risk (*n* = 76, pass rate = 40.8%), and those with a G2 above 14 as low risk (*n* = 253, pass rate = 89.7%). The chi-square test confirmed significant differences among the three groups (χ^2^(2) = 276.29, *p* < 0.001). This grading scheme demonstrates that mid-term performance effectively distinguishes students with substantially different probabilities of final success. The system also incorporates a dynamic adjustment mechanism based on family relationship scores to ensure the precision of warnings and the targeted nature of educational interventions. Table 3 demonstrates the classification effectiveness of the three-tier early-warning system. Mid-term grades serve as the primary indicator for warning. The model identified the decisive factors influencing academic outcomes; it effectively distinguished high-risk groups with a pass rate of only 10% from low-risk groups with a pass rate of 89.7%. The chi-square test supported that the risk classification exhibited high statistical significance (χ^2^(2) = 276.29, *p* < 0.001). With an overall model accuracy of 88.61%, the study validated its recognition capability and reliability in practical educational settings (Mohammed, 2024).

For high-risk student groups, it is recommended to implement a comprehensive intervention program centered on “academic emergency care,” integrating behavioral correction with systematic family support. First, provide intensive, personalized subject tutoring to address knowledge gaps identified in their mid-term performance, thereby preventing the “Matthew effect” of academic decline (Nennstiel, 2023). Second, establish a targeted attendance intervention mechanism (Eklund et al., 2022), as research demonstrates that early and proactive attendance interventions effectively improve academic trajectories (Busso & Frisancho Robles, 2023). For medium-risk students, the focus lies in implementing preventive reinforcement and dynamic monitoring, providing modular remedial courses, and establishing regular academic feedback cycles, supplemented by learning analytics for fine-tuning to ensure the precision and timeliness of interventions (Ifenthaler & Yau, 2020). For low-risk students, the goal is to maintain their outstanding performance and unlock deeper potential by encouraging participation in inquiry-based learning or peer mentoring, thereby fostering a positive peer learning culture (van Leeuwen & Janssen, 2019). These approaches necessitate the integration of a layered strategy into an institutionalized, dynamic early-warning and intervention system, thereby establishing an efficient closed-loop process of assessment, early warning, and intervention (Tretow-Fish & Khalid, 2023).

## 6. Conclusions and Prospects

### 6.1. Research Conclusions

By integrating the Conservation of Resources Theory with the perspective of the Process–Person–Context–Time model, this study finds that mathematics performance among Portuguese secondary school students is not determined by a single factor but rather the cumulative result of interactions among prior knowledge, learning behaviors, and family capital. The mid-term score G2 was identified as the strongest predictor of final academic achievement, with its impact on pass probability exhibiting an exponential increase. This suggests that G2 not only reflects students’ current academic level but also indicates whether they have reached critical milestones in competency development. Notably, the estimated association of mothers’ educational attainment with mathematics performance is not constant, ranging from −0.14 to +0.47 across subgroups. This heterogeneity is primarily moderated by both the father’s educational level and the quality of family relationships: when high maternal education coexists with poor family relationships, the point estimate is negative—a pattern tentatively consistent with the “family education paradox,” though the wide confidence interval indicates this finding requires replication. Additionally, although descriptive patterns suggested some variation across study time categories, formal testing did not confirm a statistically significant nonlinear relationship after controlling for covariates, challenging the intuitive assumption that “the longer the study time, the better.” These findings collectively indicate that the mechanisms underlying academic achievement are far more complex than those described by linear models. The family emotional environment serves as a prerequisite for the effective transformation of educational capital; without this foundation, further analysis becomes inadequate. High-quality educational resources may also be ineffective or even counterproductive.

### 6.2. Research Limitations and Prospects

This study has several limitations. First, unmeasured confounding cannot be ruled out; future research could employ instrumental variables or sensitivity analyses to strengthen causal inference. Second, the sample was drawn from only two Portuguese public schools (N = 399), limiting generalizability. Third, key variables relied on student self-reports, which may introduce social desirability bias. Fourth, family relationship quality was treated as a unitary construct, yet its subdimensions may have differential effects. Fifth, the early-warning system was evaluated on the same data used for model development, risking overfitting; external validation is needed. Sixth, G1 and G2 were treated as mediators based on the DAG and excluded from covariate adjustment; therefore, our CATE estimates reflect direct effects rather than total effects of maternal education. Seventh, the psychological mechanisms underpinning the “family education paradox” were not directly measured and remain theoretical. Despite these limitations, this study offers preliminary evidence that is consistent with the COR–PPCT framework and provides a practically applicable early-warning tool pending external validation. The DAG-based identification strategy also contributes a transparent causal structure for future research.

## Figures and Tables

**Figure 1:**
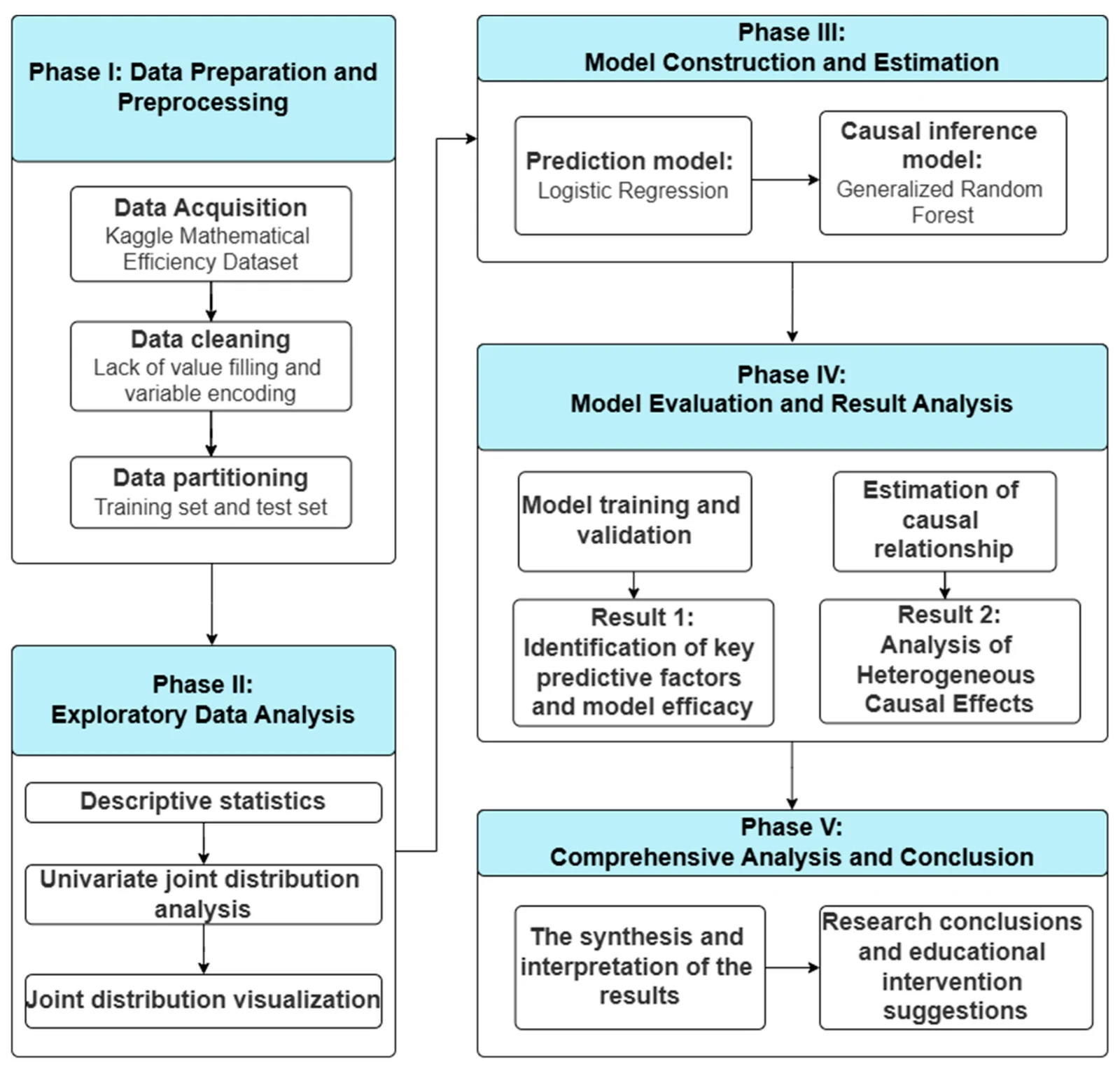
Research roadmap.

**Figure 2:**
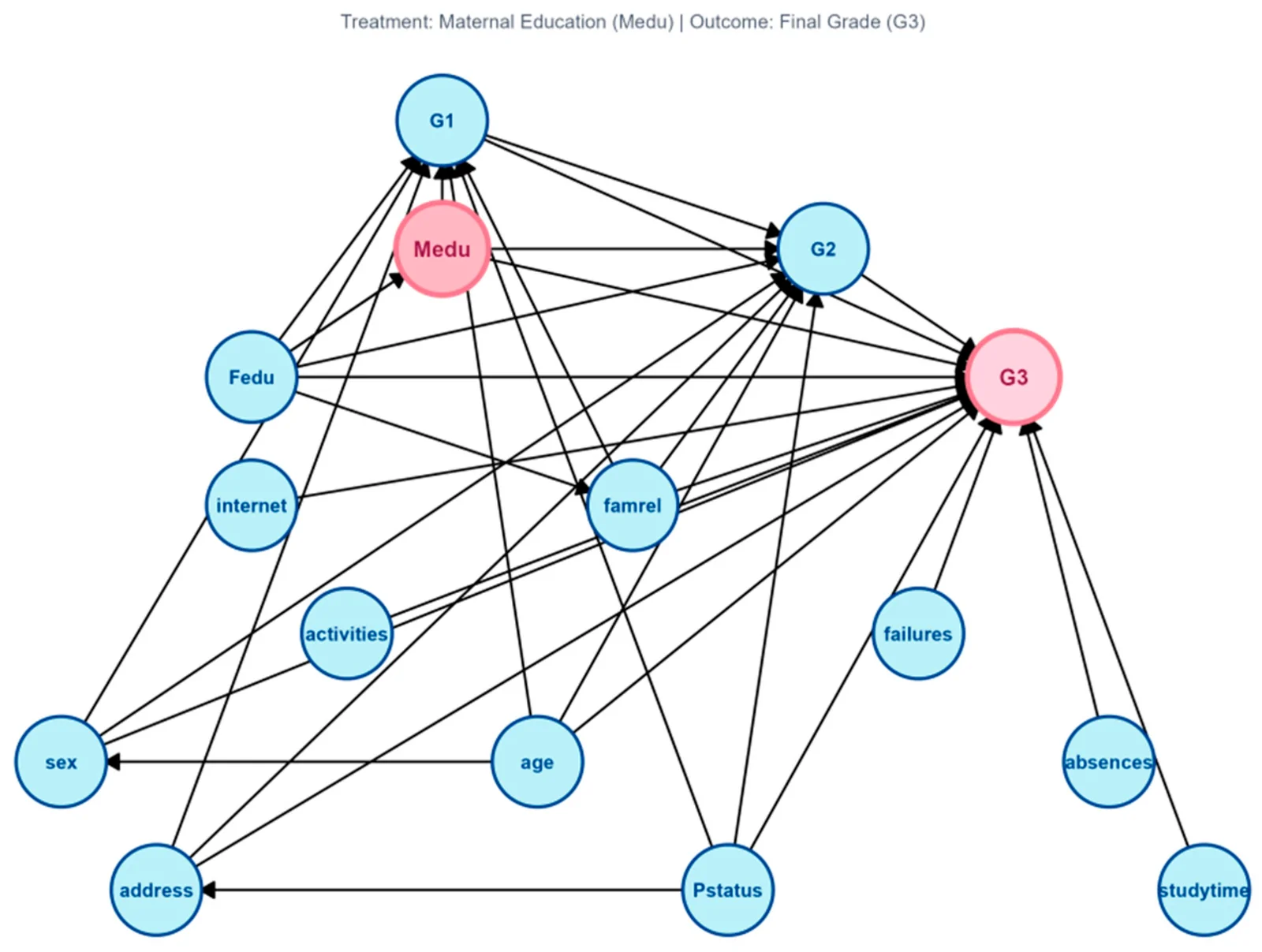
Directed acyclic graph (DAG) for causal identification. (Note: pink = treatment/outcome; blue = covariates; G1/G2 = mediators).

**Figure 3:**
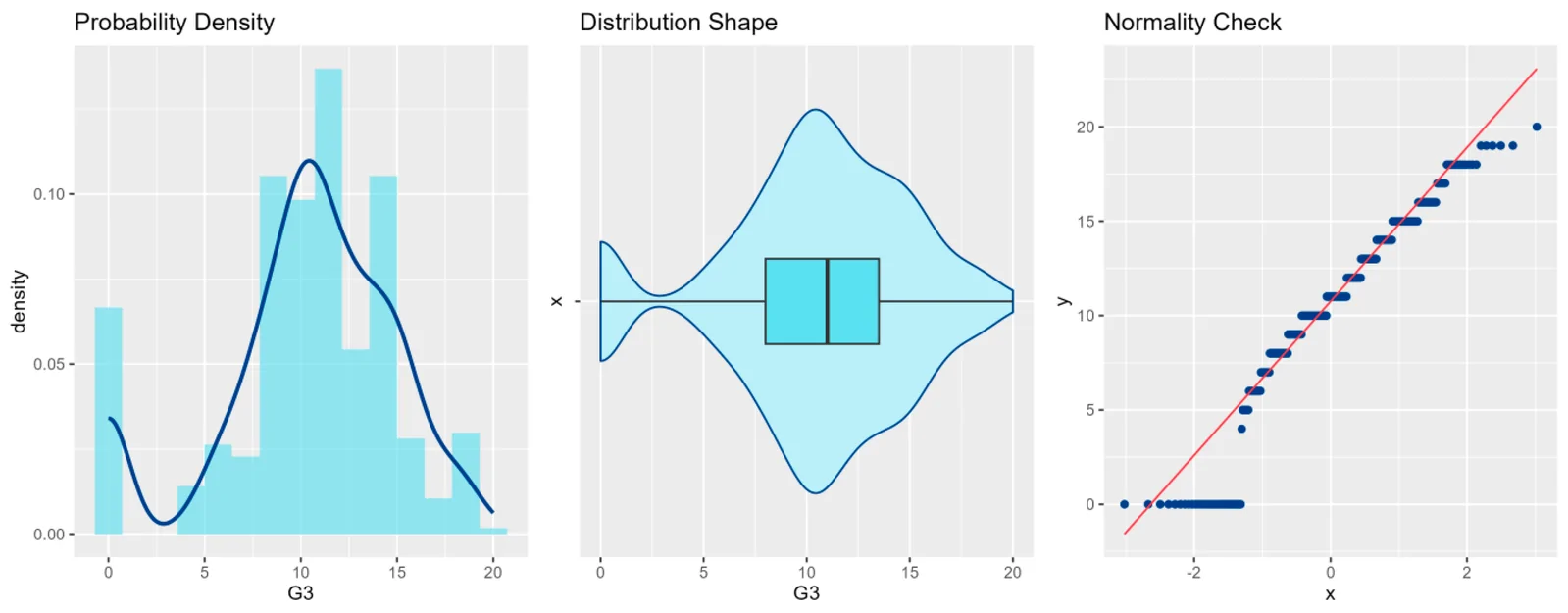
Distribution plot of final scores (G3).

**Figure 4:**
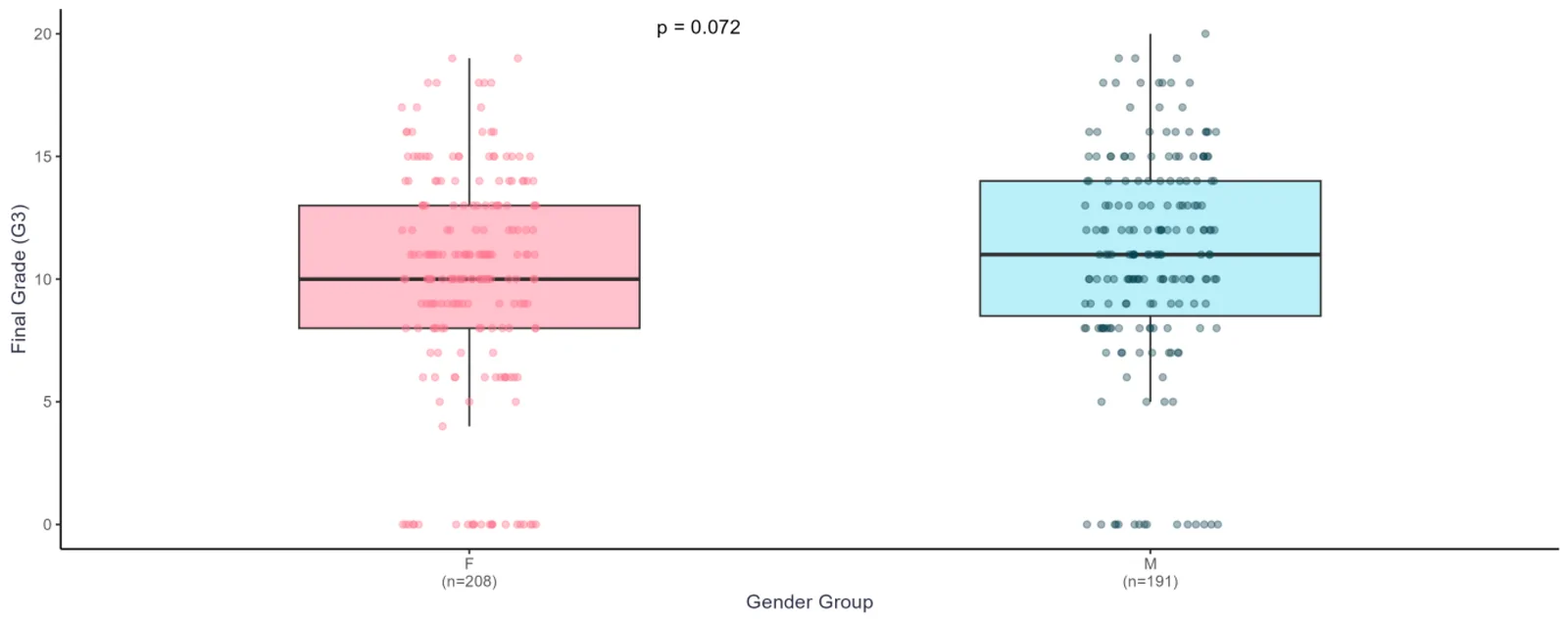
Distribution of mathematics scores among students of different genders.

**Figure 5:**
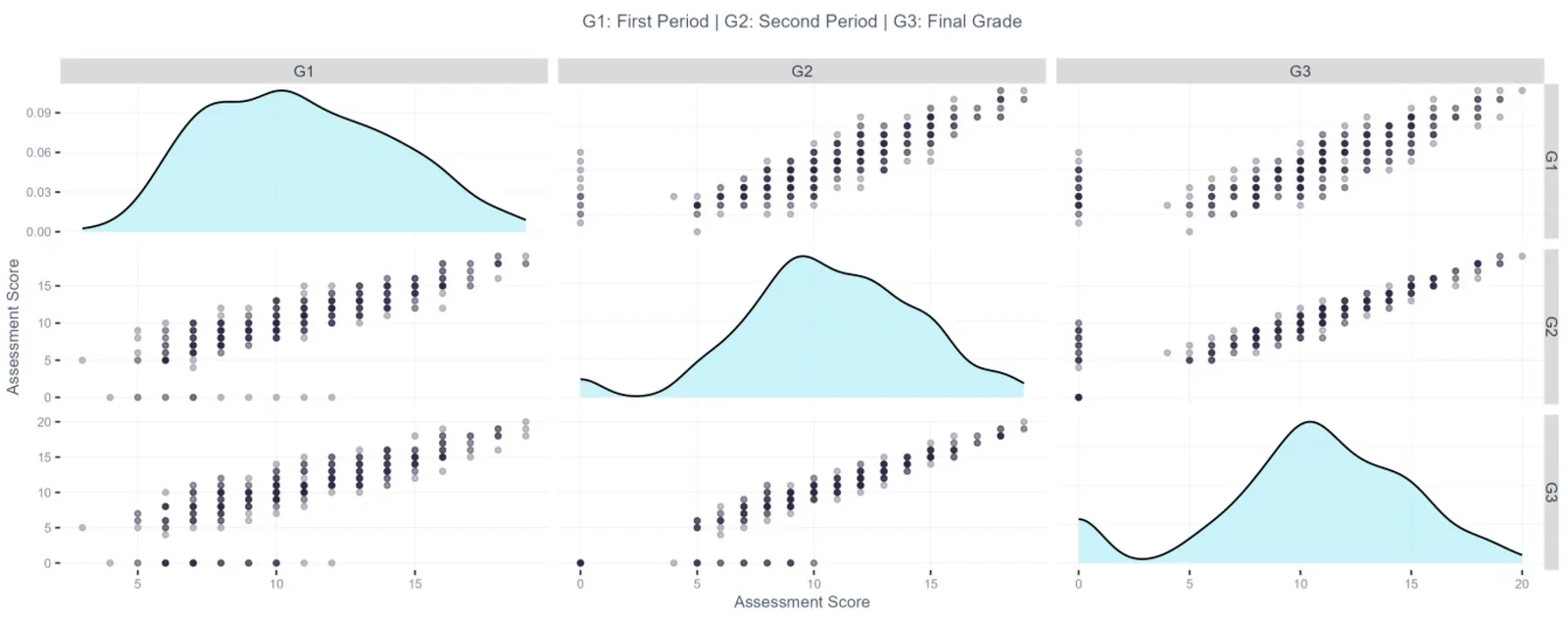
Correlation matrix diagram of the three examination scores.

**Figure 6:**
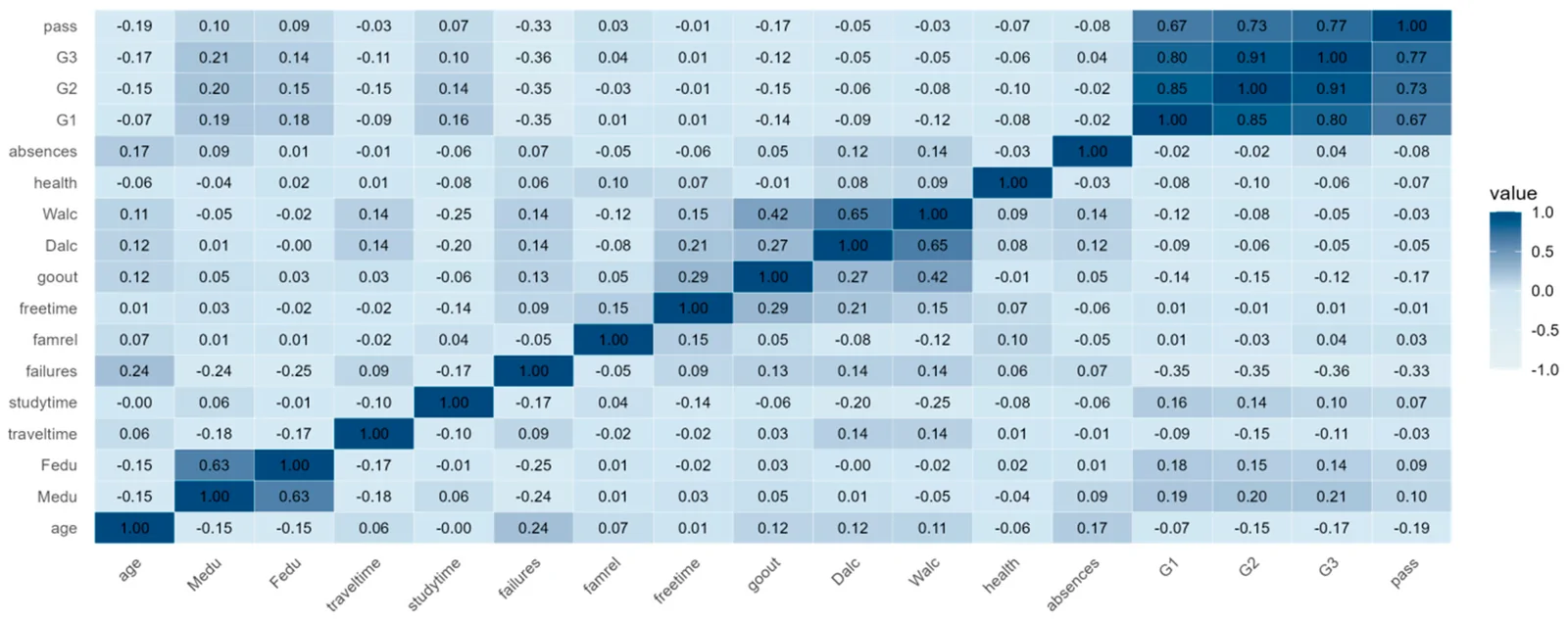
Heatmap of Pearson correlation coefficients between variables.

**Figure 7:**
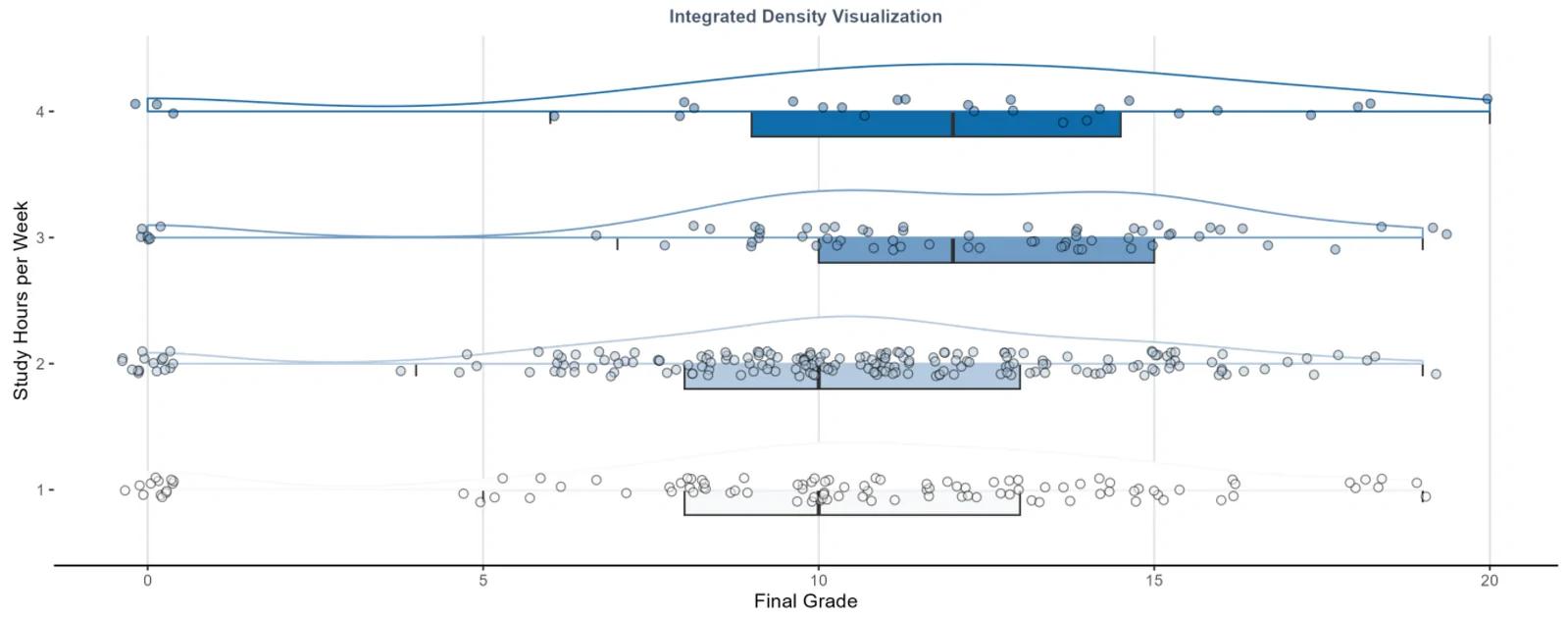
Scatter plot showing the relationship between study time and academic performance.

**Figure 8:**
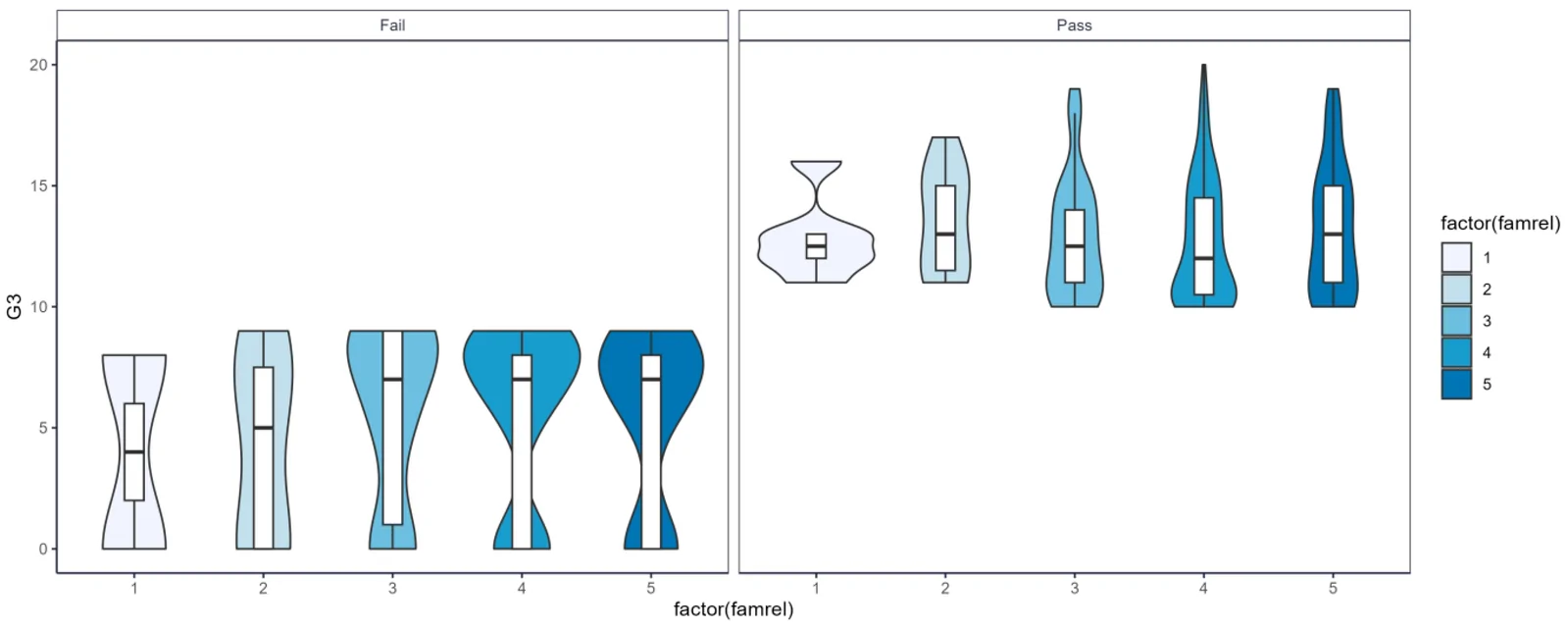
Relationship between family relationship quality and academic performance—violin diagram.

**Figure 9:**
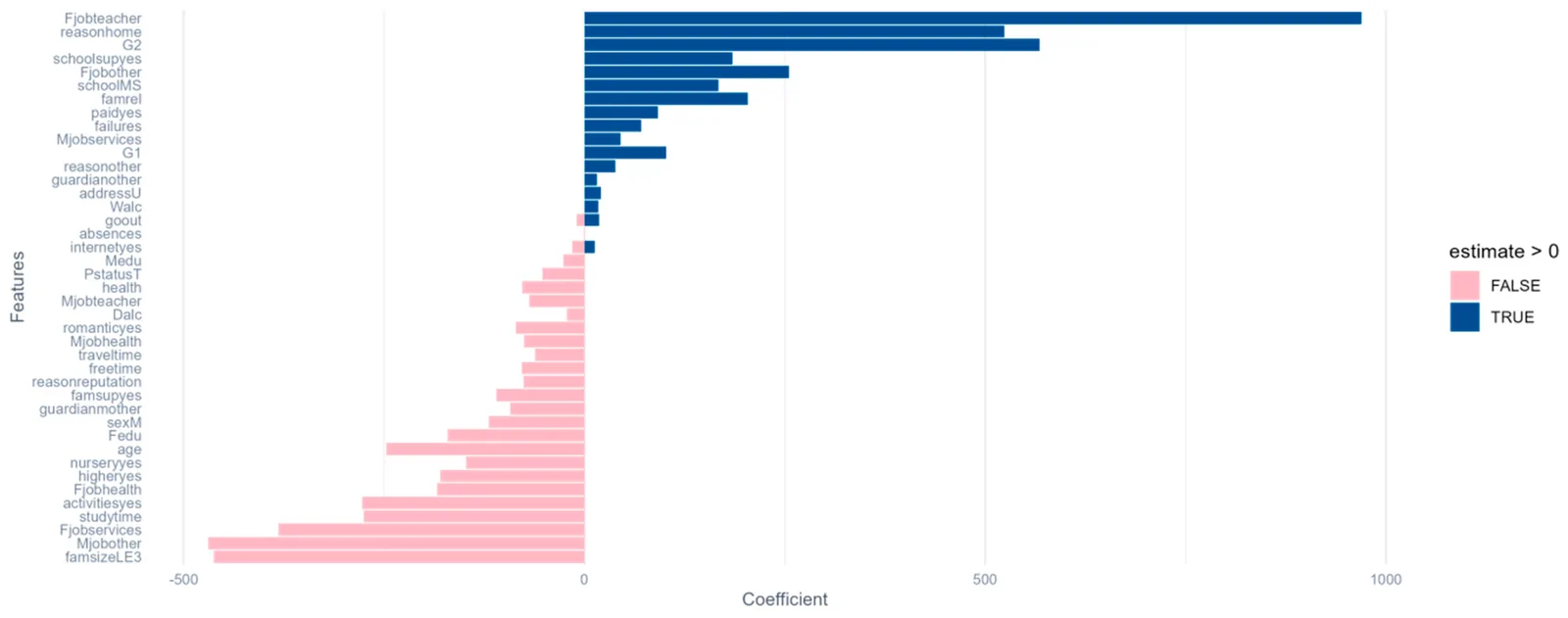
Feature coefficient plot of the logistic regression model.

**Table 1:** Definition and theoretical classification of core variables.

Variable	Operational Definition	Range	Theoretical Attribution
Maternal Education Level	Mother’s highest educational degree	1–5	Condition Resource
Paternal Education Level	Father’s highest educational degree	1–5	Exosystem Capital
Family Relationship Quality	Emotional support and communication quality	1–5	Proximal Process/Energy Resource
Initial Grade (G1)	First period grade	0–20	Resource Accumulation Node
Mid-term Grade (G2)	Second period grade	0–20	Resource Accumulation Node
Final Grade (G3)	End-of-semester summative grade	0–20	Outcome Variable
Academic Pass (Pass)	G3 ≥ 10	0/1	Outcome Variable
Number of Absences	Cumulative semester absences	Continuous	Resource Loss Indicator
Course Failure History	Number of previous course failures	Continuous	Resource Loss Indicator
Study Time	Weekly study hours	Continuous	Resource Input Indicator

**Table 2:** Heterogeneous conditional effects by maternal education.

Subgroup	*n*	CATE	95% CI
Full sample	399	0.12	[−0.04, 0.29]
Medu (≤3)	264	0.09	[−0.12, 0.29]
Medu (>3)	135	0.19	[−0.08, 0.47]
Fedu (≤3)	299	0.07	[−0.12, 0.27]
Fedu (>3)	100	0.27	[−0.02, 0.56]
Famrel (≤3)	94	−0.08	[−0.41, 0.25]
Famrel (>3)	305	0.19	[−0.01, 0.38]
Medu (≤3) + Famrel (>3)	204	0.13	[−0.12, 0.37]
Medu (≤3) + Famrel (≤3)	60	−0.04	[−0.39, 0.30]
Medu (>3) + Famrel (>3)	101	0.31	[0.02, 0.59]
Medu (>3) + Famrel (≤3)	34	−0.14	[−0.82, 0.53]
Medu (≤3) + Fedu (≤3)	238	0.05	[−0.18, 0.27]
Medu (≤3) + Fedu (>3)	26	0.47	[−0.04, 0.91]
Medu (>3) + Fedu (≤3)	61	0.19	[−0.24, 0.62]
Medu (>3) + Fedu (>3)	61	0.20	[−0.16, 0.55]

Note: CATE = expected ΔG3 per one-category increase in Medu (1–5 scale). For Medu, Fedu, and Famrel, values >3 = above-median; ≤3 = at/below-median. Some CIs are wide due to small subgroup sizes; interpret as exploratory. For subgroups defined by Medu itself, CATE estimates the expected association of moving from the current level to the next category.

**Table 3:** Effect analysis table of the three-level academic early-warning system. Test set (threshold = 0.5).

Category	Item	Statistic
Logistic Regression Coefficients	(Intercept)	Coefficient: −20.6
Logistic Regression Coefficients	G1	Coefficient: 0.164
Logistic Regression Coefficients	G2	Coefficient: 1.84
Logistic Regression Coefficients	Absences	Coefficient: −0.0333
Logistic Regression Coefficients	Famrel	Coefficient: 0.642
Three-Level Early-Warning Effect	High Risk	Count: 70, Pass Rate: 10%
Three-Level Early-Warning Effect	Medium Risk	Count: 76, Pass Rate: 40.8%
Three-Level Early-Warning Effect	Low Risk	Count: 253, Pass Rate: 89.7%
Statistical Test Results	Chi-square Test	χ^2^(2) = 276.29
Statistical Test Results	High Risk Group Baseline Test	*p* = 0.0001
Model Performance Metrics	Accuracy	88.61%
Model Performance Metrics	Sensitivity	86.79%
Model Performance Metrics	Specificity	92.31%
Model Performance Metrics	F1-Score	0.911
Model Performance Metrics	ROC-AUC	0.895

## Data Availability

The data presented in this study are available in the UCI Machine Learning Repository at https://archive.ics.uci.edu/dataset/320/student+performance (accessed on 9 January 2026), reference number 320. These data were derived from the following resources available in the public domain: Student Performance Dataset, UCI Machine Learning Repository.
